# Change in the Clinical Picture of Hospitalized Patients with COVID-19 between the Early and Late Period of Dominance of the Omicron SARS-CoV-2 Variant

**DOI:** 10.3390/jcm12175572

**Published:** 2023-08-26

**Authors:** Robert Flisiak, Dorota Zarębska-Michaluk, Krystyna Dobrowolska, Marta Rorat, Magdalena Rogalska, Justyna Anna Kryńska, Anna Moniuszko-Malinowska, Piotr Czupryna, Dorota Kozielewicz, Jerzy Jaroszewicz, Katarzyna Sikorska, Agnieszka Bednarska, Anna Piekarska, Piotr Rzymski

**Affiliations:** 1 Department of Infectious Diseases and Hepatology, Medical University of Białystok, 15-540 Białystok, Poland; robert.flisiak1@gmail.com (R.F.); pmagdar@gmail.com (M.R.); juskry4@gmail.com (J.A.K.); 2 Department of Infectious Diseases and Allergology, Jan Kochanowski University, 25-317 Kielce, Poland; dorota1010@tlen.pl; 3 Collegium Medicum, Jan Kochanowski University, 25-317 Kielce, Poland; 4 Department of Infectious Diseases and Hepatology, Wrocław Medical University, 51-149 Wrocław, Poland; marta.rorat@gmail.com; 5 Department of Forensic Medicine, Wrocław Medical University, 50-367 Wrocław, Poland; 6 Department of Infectious Diseases and Neuroinfections, Medical University of Białystok, 15-809 Białystok, Poland; anna.moniuszko@umb.edu.pl (A.M.-M.); piotr.czupryna@umb.edu.pl (P.C.); 7 Department of Infectious Diseases and Hepatology, Faculty of Medicine, Collegium Medicum in Bydgoszcz, Nicolaus Copernicus University, 87-100 Toruń, Poland; d.kozielewicz@wsoz.pl; 8 Department of Infectious Diseases and Hepatology, Medical University of Silesia in Katowice, 41-902 Bytom, Poland; jerzy.jr@gmail.com; 9 Division of Tropical and Parasitic Diseases, Faculty of Health Sciences, Medical University of Gdańsk, 80-210 Gdańsk, Poland; ksikorska@gumed.edu.pl; 10 Department of Adult’s Infectious Diseases, Medical University of Warsaw, Hospital for Infectious Diseases, 02-091 Warsaw, Poland; agnieszka.bednarska@wum.edu.pl; 11 Department of Infectious Diseases and Hepatology, Medical University of Łódź, 90-419 Łódź, Poland; annapiekar@gmail.com; 12 Department of Environmental Medicine, Poznań University of Medical Sciences, 60-806 Poznań, Poland; rzymskipiotr@ump.edu.pl

**Keywords:** SARS-CoV-2, COVID-19, Omicron, mortality, severity

## Abstract

This study aimed to compare the clinical picture of COVID-19 in the initial and later period of Omicron dominance and to identify populations still at risk. A retrospective comparison of the clinical data of 965 patients hospitalized during the early period of Omicron’s dominance (EO, January–June 2022) with 897 patients from a later period (LO, July 2022–April 2023) from the SARSTer database was performed. Patients hospitalized during LO, compared to EO, were older, had a better clinical condition on admission, had a lower need for oxygen and mechanical ventilation, had less frequent lung involvement in imaging, and showed much faster clinical improvement. Moreover, the overall mortality during EO was 14%, higher than that in LO—9%. Despite the milder course of the disease, mortality exceeding 15% was similar in both groups among patients with lung involvement. The accumulation of risk factors such as an age of 60+, comorbidities, lung involvement, and oxygen saturation <90% resulted in a constant need for oxygen in 98% of patients, an 8% risk of mechanical ventilation, and a 30% mortality rate in the LO period. Multiple logistic regression revealed lower odds of death during the LO phase. Despite the milder course of infections caused by the currently dominant subvariants, COVID-19 prophylaxis is necessary in people over 60 years of age, especially those with comorbidities, and in the case of pneumonia and respiratory failure.

## 1. Introduction

In over three years of the coronavirus disease 2019 (COVID-19) pandemic, five severe acute respiratory syndrome coronavirus 2 (SARS-CoV-2) variants of concern (VoCs) responsible for successive waves of epidemics worldwide have been identified [1]. Chronologically, the most recent is Omicron (B.1.1.529), which was designated a VoC by the World Health Organization [2] on November 2021, just days after being detected in South Africa and Botswana [3]. The emergence of this line has changed the trajectory of the COVID-19 pandemic as, despite being more transmissible predominantly due to high evasion of adaptive humoral immunity, it causes a milder course of the disease and lower mortality compared to the previous viral variants, attributed to its intrinsic biological features, such as the preference to use the endosomal pathway of cellular entry [4,5,6,7]. Selective advantages of the Omicron variant have allowed it to quickly replace the previous Delta lineage and become the dominant SARS-CoV-2 variant. Omicron spread rapidly in many regions and, due to point mutations and recombination events, gradually evolved into numerous sublines, some of which have become dominant worldwide [5,8]. Despite the lower clinical relevance of Omicron, health systems in many countries were overwhelmed by the surge in infections during the wave of this SARS-CoV-2 variant, with dynamics much higher than during previous pandemic periods [9].

According to data provided by the WHO, Omicron caused about two-thirds of the more than 768 million confirmed cases of SARS-CoV-2 infection as of 26 June 2023, and was responsible for one-fifth of the nearly 7 million deaths recorded to that date [2]. However, one should note that the true number of cases and deaths is likely significantly higher due to underdiagnosis, underreporting, and excess mortality. Most cases of COVID-19 have been reported in Europe. On this continent, Omicron began to dominate in early 2022, with the BA.1 and BA.2 subvariants prevailing during the initial period, before being replaced by BA.5, BA.2.75, and recombinant sublines BQ.1 and XBB.1.5 in the second half of 2022 [1,10,11]. All Omicron subvariants show the ability to evade neutralization efficacy induced by vaccination or past SARS-CoV-2 infection, with sublines with more enhanced immune-evasive properties emerging over time [5,12].

Numerous reports have documented a less severe course of the disease, a lower rate of hospitalization and intensive care unit (ICU) admissions, and a decrease in the mortality rate during the Omicron wave as compared to surges caused by previous variants of SARS-CoV-2 [13,14]. What is lacking, however, are studies evaluating the variability in the clinical presentation and the outcomes of COVID-19 within Omicron alone, the line responsible for the longest-lasting wave of the pandemic, and no analysis comparing the early and late phases of the Omicron wave has been published to date.

Therefore, the aim of this study was to compare the clinical picture of COVID-19 in the early and later periods of Omicron dominance and to identify populations still at risk of a severe course and outcome of the disease. This is particularly important as SARS-CoV-2 is far from eradication, continues to evolve, and requires monitoring, also using real-world clinical analyses. They are also pivotal as a reference point for future assessment of the clinical relevance of SARS-CoV-2 (sub)variants yet to come, particularly given the fact that the World Health Organization announced on 5 May 2023 that COVID-19 is no longer considered a Public Health Emergency of International Concern but remains an established and ongoing health issue [15,16].

## 2. Materials and Methods

**Figure 1:**
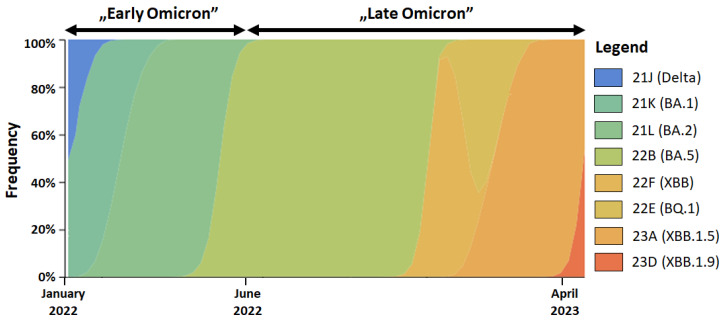
The dominant Omicron lineage subvariants in Poland during two periods defined for the purpose of this study as early Omicron and late Omicron waves. The data and graphs retrieved from Nexstrain.org [17].

The data for this study were collected retrospectively, using the observational, multicenter, nationwide SARSTer database supported by the Polish Association of Epidemiologists and Infectiologists, which has been operating since mid-2020 and includes data from 13,632 adult patients hospitalized in 30 centers due to COVID-19, who were diagnosed and treated according to the national recommendations [17,18]. Following these guidelines, patients were evaluated for their coinfection with the influenza virus. For the purpose of this study, a comparison of the clinical data of 965 patients hospitalized during the early period of Omicron’s dominance (EO, January–June 2022) with 897 patients from a later period (LO, July 2022–April 2023) was performed. These were all consecutive patients hospitalized at the centers participating in the SARSTer study; the only inclusion criterion was the time of admission. The periods taken into account in the present study were established based on sequences submitted by Polish laboratories according to the Global Initiative on Sharing All Influenza Data (GISAID), the most reliable database on SARS-CoV-2 variants’ prevalence in different regions of the world [19]. According to data available for our country, the defined EO period was dominated by the Omicron subvariants BA.1 and BA.2, whereas the LO period was dominated by subvariants BA.5, BQ.1, XBB, and XBB.1.5, with a small contribution of XBB.1.9 that emerged at the end of the considered timeframe (Figure 1).

Patient characteristics included gender, age, BMI, and comorbidities. Analysis of the course of the disease was assessed within 28 days from the beginning of hospitalization, including the follow-up period after discharge. It included symptoms of the disease, respiratory function on admission to the hospital based on oxygen saturation (SpO_2_), lung changes on imaging, treatment administered, the length of hospitalization, the need for oxygen therapy and mechanical ventilation, and the frequency of death. Moreover, the clinical course of the disease was assessed on admission to the hospital, and then after 7, 14, 21, and 28 days, using an ordinal scale based on WHO recommendations, modified to the 8-point version to match the specificity of the healthcare system, which was used previously [4,13,20,21]. The score was defined as follows: (1) not hospitalized, no activity restrictions; (2) not hospitalized, no activity restrictions, and/or not requiring oxygen supplementation at home; (3) hospitalized, and not requiring oxygen supplementation and not requiring medical care; (4) hospitalized, not requiring oxygen supplementation but requiring medical care; (5) hospitalized, requiring normal oxygen supplementation; (6) hospitalized, requiring non-invasive ventilation with high-flow oxygen equipment; (7) hospitalized, for invasive mechanical ventilation or extracorporeal membrane oxygenation; and (8) death.

Statistical analyses were conducted using Statistica v. 13 (StatSoft, Tulsa, OK, USA) and MedCalc v. 15.8 (MedCalc Software Ltd., Ostend, Belgium). Differences in frequencies of events between EO and LO groups were assessed with χ^2^ Pearson’s test or Fisher exact test (when the number of observations was <10 in any compared category). The differences in data expressed on the interval scale (age, BMI, saturation) were evaluated with a non-parametric Mann–Whitney U test because they did not meet the Gaussian assumption (Shapiro–Wilk’s test, *p* < 0.05). Multiple logistic regression models were used to evaluate the association between death or the need to use mechanical ventilation and the phase of Omicron dominance. The confounding variables included in both models were age > 60 years, obesity (BMI > 30 kg/m^2^), male sex, comorbidities, baseline SpO_2_ ≤ 90% at admission, and the presence of chest imagining changes. A *p*-value of less than 0.05 was considered statistically significant in all analyses.

## 3. Results

As shown in Table 1, patients hospitalized in the LO phase were significantly older (mean ± SD; 73.7 ± 14.8) than those in the EO phase (68.7 ± 18.2), and the proportion of patients ≥60 years of age was significantly higher in the LO phase than in the EO phase (86% vs. 74%). In the LO phase, the percentages of patients with hypertension (65.9%) and ischemic heart disease (27.1%) were significantly higher than in the early phase (57.2% and 20.7%, respectively). No cases of coinfection with influenza virus were reported in the analysed subpopulations.

The proportion of patients with severe respiratory failure (SpO_2_ ≤ 90%) decreased significantly in the LO phase compared to the EO phase (23.7% vs. 30.4%), and the mean SpO_2_ value was significantly higher during the LO period (Table 2). In the LO phase, compared to the EO phase, the percentage of patients with olfactory disorders decreased (0.7% vs. 2.6%), and the percentage of patients with fatigue (39.1% vs. 34.1%) and fever (56.4% vs. 48.7%) increased. However, these symptoms were definitely less frequent than in the period of dominance of the Delta variant. Lung involvement typical of COVID-19 was found less frequently in imaging studies in the LO period (42.7%) than in the EO period (58.8%) (Table 2). Antivirals, such as remdesivir, molnupiravir, and Paxlovid, were used in 53% of patients in the EO phase and 63% in the LO phase. The percentage of patients treated with all immunomodulators (dexamethasone, tocilizumab, and baricitinib) decreased significantly during LO (24% vs. 44%) (Table 2). Clinical improvement (≥2 point change on the ordinal scale) was observed in a greater proportion of patients during the LO period than during the EO period, regardless of the time point of the follow-up, but a statistically significant difference was noted only at 7 and 14 days of follow-up (Table 2).

**Figure 2:**
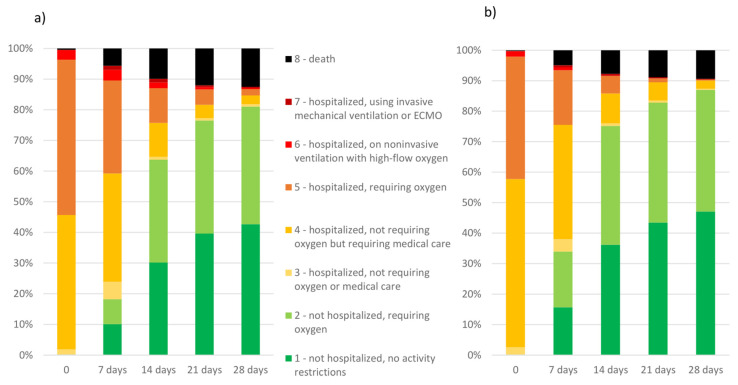
Patients’ clinical condition as classified on an ordinal scale at the time of hospitalization (0), and after 7, 14, 21, and 28 days, including the follow-up period after hospital discharge during the domination of early (**a**) and late (**b**) Omicron subvariants.

As shown in Figure 1, in the LO phase, more patients were discharged from the hospital after 7 days of hospitalization than in the EO period. In turn, the percentage of patients requiring oxygen therapy in the EO phase was higher than in the LO phase. It is especially visible in the early period of hospitalization (7, 14 days) (Figure 2).

The mean hospitalization time in the EO phase was significantly longer (11.85 ± 9.15 days) compared to the LO phase (9.34 ± 7.05 days). The percentages of patients requiring continuous oxygen therapy (47.7% vs. 38.4%), mechanical ventilation (3.0% vs. 1.5%), and death within 28 days (13.9% vs. 8.9%) were significantly more frequent in the EO period than in the LO period (Table 3). A similar trend was observed in the analysis of subpopulations of patients aged over 60 years with comorbidities or lung lesions documented by imaging. In both periods, the mortality rate in patients with inflammatory changes in the lungs exceeded as much as 15%. However, the accumulation of risk factors was associated with statistically significant differences only with regard to the need for oxygen therapy.

As shown using the logistic multiple regression models, infection during LO was associated with lower odds of death, while comorbidities and SpO_2_ < 90% at admission significantly increased these odds. In turn, the presence of imaging lung changes and SpO_2_ < 90% were independent predictors of higher odds of mechanical ventilation (Table 4).

## 4. Discussion

While each of the previous SARS-CoV-2 VoCs dominated the world during the pandemic for a few months, the current dominant Omicron line has prevailed for more than a year and a half [16]. During this time, the virus has continued to evolve and mutate, resulting in new subvariants, including recombinant ones, that have serially transitioned into globally dominant forms. A significantly higher number of mutations within the spike protein in Omicron compared to previous SARS-CoV-2 VoCs facilitates immune escape, accelerates transmission, and increases infectivity [16,22]. This results in a high number of infections, not only primary but also reinfections, even within the same lineage, which prompted the introduction of variant-adapted vaccines in late 2022 [23,24].

However, in parallel with these phenomena, a milder clinical course of COVID-19 and lower mortality caused by the Omicron variant has been documented compared to previous pandemic waves [4,13,14,25,26]. Available analyses evaluating the clinical picture and outcomes of COVID-19 focus on the comparison between infections caused by the Omicron variant with other VoCs or separately describe different periods of Omicron dominance [27,28,29,30]. There is a lack of publications comparing the severity of the clinical course of SARS-CoV-2 infection in patients hospitalized in the early and late periods of Omicron prevalence. To date, several comparative studies have been published taking into account sublines BA.1, BA.2, BA.4, and BA.5. However, to the best of our knowledge, the published data refer to the period up to mid-2022, cover a small percentage of hospitalized patients, and do not concern Europe [30,31,32,33]. The comparative study assessed the late period of the Omicron wave, also including the prevalence of the recombinant subvariants and covering the period from July 2022 to January 2023, and was conducted in India, but again, only 23% of patients were hospitalized [27]. Therefore, our study fills a knowledge gap in the area of SARS-CoV-2-infected hospitalized patients in Europe and provides a reference point for future epidemiological and real-world clinical analyses.

In the present study, the division we made between the early and late phases of the Omicron wave according to GISAID data for our country corresponds to the periods of dominance of the BA.1 and BA.2 variants, followed by BA.5, BQ.1, XBB, and XBB.1.5, respectively, and the caesura was mid-2022 [19]. We found that in LO, the mean age of hospitalized patients increased compared to the EO period, and the share of those aged 60 and older was significantly higher. Thus, we documented the continuation of the trend of the increasing age of hospitalized patients observed in the Omicron wave compared to the previous Delta surge confirmed by other researchers [26].

In the symptomatology on admission, the very low proportion of patients with olfactory and taste disorders is noteworthy, especially in LO, 0.7% vs. 2.6% in EO. At the very beginning of the pandemic, it was a typical symptom reported in a much larger percentage of patients, even exceeding 50% in some reports, and then its frequency was observed to decrease in infections with subsequent VoCs [13,34,35]. We found fever, cough, dyspnea, and fatigue to be the most common symptoms at baseline, which is consistent with other reports [27,36].

In our analysis, patients admitted to the hospital in the LO phase were in a better clinical condition and were significantly less often in an unstable state with oxygen saturation equal to or lower than 90%. Over 80% of hospitalized patients underwent lung imaging (mainly computed tomography) in both waves considered in this study, and a significantly lower percentage of lung changes in LO patients was observed. This also supports the thesis of a milder clinical course in this period. However, it should be noted that in both analyzed Omicron wave phases, this rate was relatively low compared to earlier periods of the pandemic, especially the Delta surge, supporting results from other analyses [37,38]. The lower degree of lung involvement could be explained by the fact that the viral load during infection with previous SARS-CoV-2 strains was higher in the lungs, while in infection with the Omicron variant, it is higher in the upper respiratory tract [39].

Numerous studies assessing the severity of the disease during the Omicron wave focused on the risk of hospitalization due to infection with specific variants, which is significantly lower compared to the Delta wave and reaches up to 2% [40,41]. Unlike such analyses, we did not compare SARS-CoV-2 infections in the general population but their course and outcomes in the group of hospitalized individuals, keeping in mind that any hospitalization, especially in more vulnerable patients, may be associated with the risk of severe progression, which could be related to the age and patients’ underlying condition and not only to the characteristics of the viral variant. Indeed, a higher mortality rate, regardless of the phase of the Omicron dominance, was documented in patients over 60 years of age and burdened with comorbidities, supporting findings from other reports [30,32]. However, we confirmed the greatest negative effect on mortality for the presence of inflammatory changes in the lungs in imaging examinations. It should be emphasized that although the overall mortality in the LO phase was lower, it reached as much as 30% in the group with the greatest accumulation of risk factors, being 6% higher than in the similar group during the EO period. The same parameters that were associated with higher mortality adversely affected the need for oxygen therapy, and again, in the analyzed population, it was significantly lower in the LO phase, confirming the thesis of a milder course of infection during this period. Finally, this was also confirmed by multiple regression analysis that revealed significantly decreased odds of death during LO when controlling for potential risk factors, such as age, BMI, sex, comorbidities, low saturation, and changes in chest imaging at admission.

Nevertheless, based on existing evidence, it cannot be assumed that the future evolution of SARS-CoV-2 will always lead to a decrease in the clinical relevance of infections [16,42]. Although the Omicron lineage is generally characterized by lower fusogenicity, a parameter that affects viral pathogenicity, more recent sublineages, including XBB, revealed enhanced fusogenic potential [43,44,45]. Although this may not translate into greater clinical relevance, future viral evolution could potentially drive gradually increased fusogenicity, particularly if it is accompanied by mutations leading to higher viral loads and, subsequently, better transmissibility. Under such a scenario, the odds of an overactive pro-inflammatory and cytotoxic immune response in infected individuals would be increased, leading to a higher clinical severity of COVID-19. Therefore, it continues to be necessary to ensure a high uptake of COVID-19 vaccines, including booster doses, especially in risk groups such as elderly individuals with comorbidities [16].

In addition to the potential for immune escape due to numerous mutations, the importance of the therapeutic escape of Omicron subvariants is also emphasized [8,12,29,46]. This resistance or reduced susceptibility compared to previous VoCs applies to monoclonal antibodies, while the antiviral drugs used so far remain active against recombinant subvariants of Omicron [5,8,47,48]. In our study, monoclonal antibodies were administered in single patients during the EO phase. Antivirals were used more frequently in the LO period, while the administration of immunomodulators significantly decreased from 44% in EO to 24% in the LO phase. This was related to the less frequently observed progression of the disease from the viral to the immune phase and is another confirmation of a milder course of infection and documented faster clinical improvement assessed according to the WHO ordinal scale.

Several limitations of our study must be addressed. Firstly, it had a retrospective observational design with possible bias and lacked some data. Secondly, the exact SARS-CoV-2 Omicron subvariant with which the considered patients were infected was not established using viral genome sequencing, but instead, we used reliable data from the GISAID database to distinguish two periods characterized by the domination of different Omicron sublineages. Thirdly, we did not evaluate the impact of vaccination because in order to plausibly assess the influence of this parameter, one would have to take into account not only the fact of vaccination, but the number of doses, the type of vaccine, and the time interval of COVID-19 since the last vaccination. Moreover, it appears that in the current phase of the pandemic, even in countries with insufficient vaccination rates, such as Poland, a large percentage of the population has already been naturally immunized [49]. We also did not collect data on reinfection, but given the high risk of such events in the Omicron wave [40,50], this knowledge would not have contributed much to the analysis. 

However, the strength of our study is the assessment of clinical differences and the comparison of the short-term prognosis between the two periods of the Omicron wave among a large cohort of patients hospitalized for COVID-19, as well as its multicenter nature, which allows the generalization of the results.

## 5. Conclusions

The older age of patients, better condition at hospital admission with a lower percentage of those with pneumonia on imaging, better prognosis with a less frequent need for oxygen therapy and mechanical ventilation, and lower mortality characterize the late period of Omicron dominance compared to its early phase. The decreased odds of death during this period were independent of a patient’s characteristics and clinical state at admission. However, the age of over 60 years, the presence of inflammatory changes in the lungs, and comorbidities worsened the prognosis in both periods of the Omicron wave. In these patient populations at risk of a more severe course and unfavorable outcomes of COVID-19, it is necessary to provide appropriate antiviral medication and ensure its continuous availability, promote immunization with variant-adapted vaccines, and emphasize the importance of booster vaccine doses, particularly in periods during which an increased number of SARS-CoV-2 infections can be expected.

## Figures and Tables

**Table 1:** Patients’ characteristics during the domination of early and late Omicron subvariants.

	Early Omicron (*n* = 965)	Late Omicron (*n* = 897)	*p*-Value
The average number of patients per month, *n*	161	90	-
**Demographic characteristics**			
BMI, x ±SD	27.0 ± 5.5	27.1 ± 5.5	*U* = 278,693, *z* = 0.15, *p* = 0.884
Gender, females/males, *n* (%)	494/471 (51.2/48.8)	472/425 (52.6/47.4)	χ^2^ = 0.3, *p* = 0.538
Age (years), mean ±SD	68.7 ± 18.2	73.7 ± 14.8	*U* = 370,909, *z* = −5.30, *p* < 0.001
<20 years, *n* (%)	1 (0.1)	2 (0.2)	*p* = 0.611
20–40 years, *n* (%)	106 (11.0)	36 (4.0)	χ^2^ = 32.1, *p* < 0.001
40–60 years, *n* (%)	142 (14.7)	89 (9.9)	χ^2^ = 9.8, *p* = 0.002
60–80 years, *n* (%)	416 (43.2)	452 (50.4)	χ^2^ = 9.9, *p* = 0.002
>80 years, *n* (%)	299 (31.0)	318 (35.5)	χ^2^ = 4.2, *p* = 0.04
**Comorbidities**			
Any comorbidities	889 (92.1)	828 (92.3)	χ^2^ = 0.02, *p* = 0.883
Hypertension	552 (57.2)	591 (65.9)	χ^2^ = 14.8, *p* = 0.002
Myocardial ischemic disease	200 (20.7)	243 (27.1)	χ^2^ = 10.4, *p* = 0.013
Other cardiovascular diseases	310 (32.1)	271 (30.2)	χ^2^ = 0.79, *p* = 0.373
Chronic obstructive pulmonary disease	74 (7.7)	71 (7.9)	χ^2^ = 0.04, *p* = 0.843
Other respiratory diseases	114 (11.8)	80 (8.9)	χ^2^ = 4.3, *p* = 0.039
Diabetes	239 (24.8)	233 (26.0)	χ^2^ = 0.36, *p* = 0.549
Other metabolic diseases	144 (14.9)	137 (15.3)	χ^2^ = 0.045, *p* = 0.833
Cancers	150 (15.5)	155 (17.3)	χ^2^ = 1.0, *p* = 0.312
Stroke	95 (9.8)	92 (10.3)	χ^2^ = 0.09, *p* = 0.768

BMI, body mass index; SD, standard deviation.

**Table 2:** Symptoms, respiratory function, lung changes on imaging, treatment administered, and clinical improvement in patients during the domination of early and late Omicron subvariants.

	Early Omicron (*n* = 965)	Late Omicron (*n* = 897)	*p*-Value
**Symptoms of the disease**			
Cough, *n* (%)	475 (49.2)	441 (49.2)	χ^2^ = 0.001, *p* = 0.980
Fever, *n* (%)	470 (48.7)	506 (56.4)	χ^2^ = 9.6, *p* = 0.002
Dyspnea, *n* (%)	350 (36.3)	307 (34.2)	χ^2^ = 0.851, *p* = 0.356
Disturbances of smell and/or taste, *n* (%)	25 (2.6)	6 (0.7)	*p* = 0.002
Diarrhea, *n* (%)	101 (10.5)	72 (8.0)	χ^2^ = 3.2, *p* = 0.07
Headaches, *n* (%)	89 (9.2)	99 (11.0)	χ^2^ = 1.7, *p* = 0.194
Nausea, *n* (%)	71 (7.4)	69 (7.7)	χ^2^ = 0.07, *p* = 0.784
Vomiting, *n* (%)	66 (6.8)	69 (7.7)	χ^2^ = 0.503, *p* = 0.478
Fatigue, *n* (%)	329 (34.1)	351 (39.1)	χ^2^ = 5.1, *p* = 0.02
**Respiratory function on admission to the hospital**			
SpO_2_ (%), mean ± SD	92.1 ± 5.6	92.5 ± 5.8	*U* = 376,903, *z* = −2.35, *p* < 0.001
Asymptomatic, *n* (%)	49 (5.1)	19 (2.1)	χ^2^ = 11.6, *p* < 0.001
Symptomatic stable, SpO_2_ > 95%, *n* (%)	288 (29.8)	315 (35.1)	χ^2^ = 5.9, *p* = 0.015
Symptomatic unstable, SpO_2_ 90–95%, *n* (%)	306 (31.7)	297 (33.1)	χ^2^ = 0.41, *p* = 0.519
Symptomatic unstable, SpO_2_ ≤ 90%, *n* (%)	293 (30.4)	213 (23.7)	χ^2^ = 4.3, *p* = 0.04
ARDS, *n* (%)	10 (1.0)	6 (0.7)	*p* = 0.457
Unknown, *n* (%)	19 (2.0)	47 (5.2)	χ^2^ = 14.5, *p* < 0.001
**Lung changes on imaging**			
Examinations done, *n* (%)	823 (85.3)	799 (89.1)	χ^2^ = 6.0, *p* = 0.015
Changes detected by any method, *n* (% of done)	484 (58.8)	341 (42.7)	χ^2^ = 27.8, *p* < 0.001
Changes detected in X-ray, *n* (% of done)	131 (15.9)	108 (13.5)	χ^2^ = 0.98, *p* = 0.322
Changes detected by CT *n* (% of done)	366 (44.5)	241 (30.2)	χ^2^ = 25.9, *p* < 0.001
Changes detected by ultrasound, *n* (% of done)	1 (0.1)	1 (0.1)	*p* = 1.0
**Treatment administered**			
Heparin, *n* (%)	558 (57.8)	511 (57.0)	χ^2^ = 0.139, *p* = 0.709
Remdesivir, *n* (%)	276 (28.6)	392 (43.7)	χ^2^ = 46.1, *p* < 0.001
Molnupiravir, *n* (%)	233 (24.1)	103 (11.5)	χ^2^ = 50.4, *p* < 0.001
Nirmatrelvir/ritonavir, *n* (%)	0 (0.0)	66 (7.4)	*p* < 0.001
Casirivimab, *n* (%)	21 (2.2)	0 (0.0)	*p* < 0.001
Convalescent plasma, *n* (%)	4 (0.4)	0 (0.0)	*p* = 0.126
Dexamethasone, *n* (%)	305 (31.6)	190 (21.2)	χ^2^ = 25.9, *p* < 0.001
Tocilizumab, *n* (%)	89 (9.2)	23 (2.6)	χ^2^ = 36.5, *p* < 0.001
Baricitinib, *n* (%)	29 (3.0)	1 (0.1)	*p* < 0.001
Azithromycin, *n* (%)	3 (0.3)	5 (0.6)	*p* = 0.493
**Clinical improvement (at least 2 points decrease from baseline in the ordinal scale)**			
7 days, *n* (%)	180 (18.8)	294 (34.4)	χ^2^ = 48.9, *p* < 0.001
14 days, *n* (%)	612 (63.8)	641 (75.1)	χ^2^ = 13.7, *p* = 0.002
21 days, *n* (%)	735 (76.6)	709 (83.0)	χ^2^ = 2.2, *p* = 0.137
28 days, *n* (%)	779 (81.2)	741 (86.8)	χ^2^ = 1.1, *p* = 0.294

SpO_2_, Saturation of Peripheral Oxygen; SD, standard deviation; ARDS, acute respiratory distress syndrome; CT, computed tomography.

**Table 3:** Comparison of clinical indicators of the severity of the disease (oxygen need, mechanical ventilation, death) assessed in patients during the domination of early (EO) and late Omicron (LO) subvariants depending on the presence of risk factors such as changes in the lungs, age, concomitant diseases as well as their accumulation combined with oxygen saturation.

	Number of Patients *n*		Need for Oxygen Therapy *n* (%)		Need for Mechanical Ventilation *n* (%)		Death *n* (%)	
	EO	LO	EO	LO	EO	LO	EO	LO
All patients	965	897	457 (47.4)	344 (38.4)	29 (3.0)	13 (1.5)	134 (13.9)	80 (8.9)
			χ^2^ = 15.4, ***p* < 0.001**		χ^2^ = 5.1, ***p* = 0.02**		χ^2^ = 11.3, ***p* < 0.001**	
Age >60 years (60+)	716	775	395 (55.2)	331 (42.7)	24 (3.4)	12 (1.5)	124 (17.3)	77 (9.9)
			χ^2^ = 23.1, ***p* < 0.001**		χ^2^ = 3.4, *p* = 0.06		χ^2^ = 17.4, ***p* < 0.001**	
Imaging changes (IC)	484	556	318 (65.7)	198 (58.1)	23 (4.8)	11 (3.2)	78 (16.1)	54 (15.8)
			χ^2^ = 93.7, ***p* < 0.001**		χ^2^ = 6.3, ***p* = 0.01**		χ^2^ = 9.6, ***p* = 0.002**	
Comorbidities (CM)	889	828	432 (48.6)	336 (40.6)	26 (2.9)	13 (1.6)	129 (14.5)	78 (9.4)
			χ^2^ = 11.1, ***p* < 0.001**		χ^2^ = 3.5, *p* = 0.06		χ^2^ = 10.5, ***p* = 0.001**	
**Accumulation of risk factors**								
60+/IC/CM	383	307	270 (70.5)	188 (61.2)	18 (4.7)	10 (3.3)	71 (18.5)	52 (17.7)
			χ^2^ = 6.5, ***p* = 0.01**		χ^2^ = 0.91, *p* = 0.340		χ^2^ = 0.29, *p* = 0.585	
60+/IC/CM/SpO_2_ < 95%	306	241	252 (82.4)	181 (75.1)	15 (4.9)	10 (4.2)	59 (19.3)	47 (19.5)
			χ^2^ = 4.3, ***p* = 0.04**		χ^2^ = 0.17, *p* = 0.675		χ^2^ = 0.004, *p* = 0.948	
60+/IC/CM/SpO_2_ < 90%	165	126	155 (93.9)	123 (97.6)	11 (6.9)	10 (7.9)	40 (24.2)	38 (30.2)
			*p* = 0.160		χ^2^ = 0.17, *p* = 0.678		χ^2^ = 1.27, *p* = 0.259	

IC, imaging changes; CM, comorbidities; SpO_2_, Saturation of Peripheral Oxygen; EO—early Omicron; LO—late Omicron.

**Table 4:** Logistic multiple regression results on the association between death and patient’s characteristics and phase of Omicron domination in the studied cohort (*n* = 1862).

	OR	95% CI	*p*-Value
**Predicted outcome: death**			
Age 60+	0.93	0.59–1.45	0.741
BMI > 30 m^2^/kg	0.88	0.59–1.29	0.502
Male sex	0.99	0.70–1.39	0.959
Imaging changes	1.08	0.76–1.53	0.661
Comorbidities	2.36	1.19–6.20	0.0317
SpO_2_ < 90%	2.04	1.40–2.96	0.0002
Late Omicron phase	0.55	0.39–0.79	0.0014
**Predicted outcome: mechanical ventilation**			
Age 60+	0.76	0.31–1.90	0.562
BMI > 30 m^2^/kg	0.82	0.36–1.86	0.636
Male sex	0.80	0.40–1.64	0.545
Imaging changes	2.21	1.05–4.67	0.038
Comorbidities	3.45	0.43–27.71	0.244
SpO_2_ < 90%	1.44	1.12–3.23	0.037
Late Omicron phase	0.52	0.23–1.14	0.103

OR—odds ratio; 95% CI—95% confidence interval; BMI—body mass index; SpO_2_, Saturation of Peripheral Oxygen.

## Data Availability

The data are available through the SARSTer database https://sarster.pl/panel/report/report (accessed on 16 June 2023) after obtaining the coordinator’s approval.
